# Assessment Instruments for Social Anxiety in Oral and Public Communication Among Health Sciences Students: Protocol for a Scoping Review

**DOI:** 10.2196/93700

**Published:** 2026-06-29

**Authors:** Guilherme Ribeiro Constancio, Emerson Roberto dos Santos, João Daniel de Souza Menezes, Matheus Querino da Silva, Natália Almeida de Arnaldo Silva Rodriguez Castro, Ana Maria Rita Pedroso Vilela Torres de Carvalho Engel, Janaína Aparecida de Sales Floriano, Camila Bortoluci de Lima, Jéssica da Silva Francelino, Ronize Aparecida Domingues de Almeida Prado, Pedro Belchior da Silveira Junior, Daniele Nunes Longhi Aleixo, Franciane Michele da Silva, Denise Iglesias Lima Silvestrini, Alana Clara Santos Rocha, Renato Mendonça Ribeiro, Nádia Antônia Aparecida Poletti, Josimerci Ittavo Lamana Faria, Maysa Alahmar Bianchin, Fernando Nestor Facio Júnior, Fabiana de Souza Orlandi, Maria Helena Pinto, Rita de Cássia Helú Mendonça Ribeiro, Gerardo Maria de Araújo Filho, Júlio César André

**Affiliations:** 1 São José do Rio Preto Medical School - FAMERP Center for Studies and Development of Health Education – CEDES São José do Rio Preto, São Paulo Brazil; 2 Universidade Federal de São Carlos São Carlos, São Paulo Brazil

**Keywords:** social anxiety, oral communication, public speaking, health sciences students, psychometric validation, scoping review, academic performance

## Abstract

**Background:**

Proficiency in interpersonal communication and social skills is fundamental for health sciences students, but public speaking anxiety and communication apprehension frequently compromise academic performance, emotional well-being, and the acquisition of essential competencies. The postpandemic educational environment has exacerbated student stress, highlighting the urgent need for reliable assessment tools for social anxiety in this population.

**Objective:**

This scoping review protocol aims to map instruments used to assess public speaking anxiety and communication apprehension among health sciences students, analyzing their psychometric properties, cultural adaptations, and application contexts.

**Methods:**

Following the Joanna Briggs Institute framework and PRISMA-ScR (Preferred Reporting Items for Systematic Reviews and Meta-Analyses extension for Scoping Reviews) guidelines, a systematic search will be conducted across PubMed, Scopus, Embase, LILACS, Web of Science, the Cochrane Library, and the *Biblioteca Virtual em Saúde* Portal. Eligible studies will include those evaluating social or performance anxiety in undergraduate or postgraduate health sciences students, using validated or culturally adapted instruments. Two independent reviewers will screen studies, with discrepancies resolved by a third reviewer. Data extraction will be conducted systematically, focusing on structural and content variables. Analysis will use a mixed methods approach, combining descriptive statistics with thematic synthesis and incorporating psychometric expertise.

**Results:**

As of May 2026, protocol registration has been completed via the Open Science Framework. Systematic database searches and study selection are scheduled to commence in June 2026, with anticipated completion by December 2026, aligned with a 2-year implementation road map designed to generate comprehensive instrument mapping and secondary analyses for evidence-based health professional training.

**Conclusions:**

This protocol establishes a transparent and reproducible methodological plan, ensuring scientific rigor through adherence to Joanna Briggs Institute and PRISMA-ScR standards. It will deepen understanding of social anxiety assessment in oral communication among health sciences students and support the development of more effective interventions.

**Trial Registration:**

Open Science Framework 10.17605/OSF.IO/YQ4KM; https://osf.io/yq4km

**International Registered Report Identifier (IRRID):**

PRR1-10.2196/93700

## Introduction

Interpersonal communication and social skills are fundamental elements for both professional and academic development, particularly in the health sciences. Recent studies have demonstrated that the quality of social interactions not only influences academic performance but also significantly impacts students’ psychological well-being [1]. However, within the spectrum of social interactions, public speaking anxiety (PSA) and communication apprehension (CA) emerge as distinct barriers that specifically impede the communicative efficacy of future health professionals [2,3].

The transition to university life represents a particularly challenging period, during which many students experience elevated levels of PSA and CA. Current research indicates that approximately 32% of medical students and 28% of nursing students report significant symptoms of anxiety related to evaluative communication during their academic training [4]. More specifically, recent meta-analytical data suggest that the prevalence of high CA in health-related majors can reach up to 45%, significantly exceeding the rates observed in the general population [5,6].

The impact of communicative distress within academic settings has become even more relevant in the postpandemic context. Recent studies have revealed a notable increase in stress and anxiety levels among university students, with particular emphasis on situations involving oral presentations and group interactions [7]. This phenomenon is increasingly categorized through the lens of PSA, defined as the fear of speaking in front of an audience, and CA, which represents a broader, trait-like or situational anxiety associated with real or anticipated communication with others [8].

Communication and public speaking skills are especially critical for health professionals and are considered essential competencies for effective practice. However, contemporary research indicates that more than 45% of students in the health field report significant difficulties in public speaking or assertive communication situations [9]. The distinction between these constructs is vital: although PSA is often task specific, CA can pervade various interpersonal contexts, including patient–health care professionals interactions and interprofessional collaboration [10].

Excessive stress associated with PSA and CA may lead to substantial cognitive and emotional changes, including reduced attention, concentration, and short-term memory [11]. These negative effects can compromise not only academic performance but also the development of essential professional competencies required for clinical safety and patient rapport.

Research suggests that maladaptive coping strategies, such as avoidance behaviors, may exacerbate PSA and CA symptoms [12]. The ineffectiveness of certain coping mechanisms may stem from methodological limitations or diagnostic imprecision in the instruments initially used to identify anxiety specifically in oral communication settings. Traditional social anxiety scales often lack the granularity required to differentiate between generalized social phobia and the specific communicative fears inherent in PSA and CA [13].

Although certain studies have undertaken the adaptation and validation of assessment instruments for contemporary educational contexts, such as remote learning environments, these efforts frequently exhibit methodological limitations—most notably, the reliance on nonprobabilistic sampling techniques and restriction to culturally narrow populations, which significantly constrain the generalizability and external validity of their findings [14].

The absence of culturally and psychometrically validated instruments specifically designed to assess PSA and CA in oral communication contexts reveals a critical gap in both the scientific literature and institutional support practices. Consequently, the scope of this scoping review is specifically focused on mapping instruments that measure PSA and CA, rather than broader social anxiety constructs. A scoping review on this topic may allow a comprehensive mapping of existing tools that measure these specific constructs in academic speaking situations [15].

This review adopts a thematic framework that conceptualizes oral communication as a transversal competency integral to health education, encompassing affective, behavioral, and pedagogical domains. Accordingly, a comprehensive understanding of this phenomenon necessitates a multidimensional analytical approach, one that not only considers communicative proficiency but also critically examines the emotional and psychosocial constraints encountered by students across diverse health-related academic programs.

A preliminary search was conducted in databases such as PubMed, Scopus, the Cochrane Library, and Open Science Framework (OSF) to identify existing scoping or systematic reviews on the topic. No reviews were found that comprehensively addressed instruments for assessing PSA and CA in oral communication among health sciences students, which supports the originality and relevance of this protocol.

It is anticipated that the outcomes of this scoping review will enhance the precision of PSA and CA assessment in educational contexts, thereby informing the development of pedagogical and intervention strategies that are not only evidence based but also contextually sensitive, theoretically grounded, and aligned with the principle of integrality in health professional training.

## Methods

### Study Design

This document formalizes the protocol for a scoping review, functioning as a detailed blueprint that precedes and guides the execution of the scoping review itself. The scoping review methodology was selected over other types of systematic reviews due to its capacity to comprehensively map and synthesize the existing literature on a broad and complex topic [16,17]. This approach is particularly suitable for identifying the extent, scope, and nature of available research, as well as for highlighting knowledge gaps and areas requiring future investigation. This protocol has been registered with the OSF [18].

In the context of this review, the choice of a scoping approach is justified by the expected heterogeneity of studies and assessment instruments for social anxiety in oral communication among health sciences students. A traditional systematic review, which aims to answer a more restricted question through quantitative synthesis of homogeneous results, would be inadequate for the initial mapping and characterization of the diversity of instruments, psychometric properties, and application contexts [15-17]. The scoping review, as advocated by the Joanna Briggs Institute framework, will allow a panoramic understanding of the field, characterizing available instruments, their particularities, and limitations related to their validation and cultural adaptation [19-21].

### Research Question and the Population, Concept, and Context Model

The research question was meticulously formulated using the population, concept, and context model, as recommended by the Joanna Briggs Institute for scoping reviews [21], to ensure adequate clarity, specificity, and comprehensiveness (Table 1).

**Table 1:** Definition of the guiding question.

Elements	Description
Population	Undergraduate and postgraduate students enrolled in health sciences programs (eg, nursing, psychology, medicine, physiotherapy, speech-language pathology, and dentistry)
Concept	Instruments used to assess social anxiety, social phobia, performance anxiety, or fear of public speaking, specifically in oral communication contexts
Context	Academic and health professional training contexts, including situations such as oral presentations, seminars, group discussions, clinical simulations, and interactions with real or simulated patients

The guiding question is “What instruments have been used to assess social anxiety related to oral communication and public speaking in health sciences students in academic contexts, and what are their main psychometric properties, application contexts, and levels of validation?”

### Objectives

#### General Objective

The general objective of this study is to map the instruments used to assess social anxiety related to oral communication and public speaking among health sciences students, analyzing their psychometric properties, cultural adaptations, and application contexts.

#### Specific Objectives

The specific objectives of this study are as follows:

- Identifying and characterizing existing instruments for the assessment of social anxiety in oral and public communication within the target population
- Analyzing the psychometric properties (eg, reliability and validity) of the identified instruments
- Describing the academic and educational contexts in which these instruments are applied
- Verifying the existence and nature of cultural and linguistic adaptations of the instruments
- Identifying gaps in the literature concerning the validation and application of these instruments

### Search Strategy

The search strategy was developed in collaboration with a specialized health sciences librarian. The approach used a judicious combination of controlled vocabularies (Medical Subject Headings [MeSH] from the National Library of Medicine and Health Sciences Descriptors from the Latin American and Caribbean Center on Health Sciences Information, a specialized center of the Pan American Health Organization and the World Health Organization) and free-text terms. This combination aims to maximize search sensitivity and ensure the capture of a wide range of relevant terminology. The selected electronic databases were chosen for their comprehensive coverage of the health sciences literature.

To ensure comprehensive coverage of the search strategy and to avoid missing relevant studies that may not explicitly use psychometric terminology, the search terms were developed to include broader conceptual frameworks alongside specific psychometric terms. This approach captures studies that use relevant assessment instruments even when they do not use explicit psychometric language in titles, abstracts, or keywords. Therefore, in addition to terms such as “psychometrics,” “validation,” and “questionnaires,” the strategy includes related terms such as “assessment,” “measurement,” “evaluation,” “instrument,” and “tool,” thereby maximizing search sensitivity and ensuring the identification of a comprehensive range of relevant literature.

To ensure complete reproducibility of the search methodology, the final and complete search strategies for each of the 7 selected electronic databases are presented in Table 2. These strategies include all search terms, Boolean operators, and database-specific field tags, as well as the planned search execution date of June 15, 2026. The search strategies were developed in collaboration with a specialized health sciences librarian and will be executed without temporal restrictions. The results obtained from this comprehensive search will form the foundation for subsequent screening and data extraction phases of the study selection and data collection process, as described later.

**Table 2:** Search strategy.

Databases	Search strategy^a^	Date of search
PubMed	((“Social Anxiety”[MESH] OR “Anxiety, Social”[MESH] OR “performance anxiety”[TIAB] OR “public speaking”[TIAB] OR “communication apprehension”[TIAB])) AND ((“Students, Health Occupations”[MESH] OR “medical students”[TIAB] OR “nursing students”[TIAB] OR “psychology students”[TIAB] OR “health sciences students”[TIAB] OR “university students”[TIAB])) AND ((“psychometrics”[MESH] OR “validation studies”[MESH] OR “assessment instruments”[TIAB] OR “questionnaires”[TIAB] OR “scales”[TIAB]) OR (“assessment”[TIAB] OR “measurement”[TIAB] OR “evaluation”[TIAB] OR “tool”[TIAB] OR “instrument”[TIAB] OR “rating”[TIAB] OR “clinical interview”[TIAB] OR “observation”[TIAB]) OR (“oral presentations”[TIAB] OR “seminars”[TIAB] OR “clinical practice”[TIAB] OR “simulations”[TIAB] OR “student anxiety”[TIAB] OR “classroom anxiety”[TIAB] OR “examination anxiety”[TIAB]))	June 20, 2026
Scopus	(TITLE-ABS-KEY(“social anxiety” OR “performance anxiety” OR “public speaking” OR “communication apprehension”)) AND (TITLE-ABS-KEY(“health students” OR “medical students” OR “nursing students” OR “psychology students” OR “university students”)) AND (TITLE-ABS-KEY(“psychometric properties” OR “validation” OR “assessment instrument” OR “questionnaire” OR “scale” OR “assessment” OR “measurement” OR “evaluation” OR “tool” OR “instrument” OR “rating” OR “clinical interview” OR “observation” OR “oral presentations” OR “seminars” OR “clinical practice” OR “simulations” OR “student anxiety” OR “classroom anxiety” OR “examination anxiety”))	June 20, 2026
Embase	(“social anxiety”/EXP OR “performance anxiety” OR “public speaking” OR “communication apprehension”) AND (“health science student”/EXP OR “medical student”/EXP OR “nursing student”/EXP OR “psychology student”/EXP OR “university student”) AND (“psychometrics”/EXP OR “validation study”/EXP OR “assessment tool” OR “questionnaire” OR “scale” OR “assessment” OR “measurement” OR “evaluation” OR “instrument” OR “rating” OR “clinical interview” OR “observation” OR “oral presentation” OR “seminar” OR “clinical practice” OR “simulation” OR “student anxiety” OR “classroom anxiety” OR “examination anxiety”)	June 20, 2026
Cochrane library	([mh “Social Anxiety”] OR “performance anxiety”:TI,AB,KW OR “public speaking”:TI,AB,KW OR “communication apprehension”:TI,AB,KW) AND ([mh “Students, Health Occupations”] OR “medical students”:TI,AB,KW OR “nursing students”:TI,AB,KW OR “psychology students”:TI,AB,KW OR “health sciences students”:TI,AB,KW OR “university students”:TI,AB,KW) AND ([mh “psychometrics”] OR [mh “validation studies”] OR “assessment instruments”:TI,AB,KW OR “questionnaires”:TI,AB,KW OR “scales”:TI,AB,KW OR “assessment”:TI,AB,KW OR “measurement”:TI,AB,KW OR “evaluation”:TI,AB,KW OR “tool”:TI,AB,KW OR “instrument”:TI,AB,KW OR “rating”:TI,AB,KW OR “clinical interview”:TI,AB,KW OR “observation”:TI,AB,KW OR “oral presentations”:TI,AB,KW OR “seminars”:TI,AB,KW OR “clinical practice”:TI,AB,KW OR “simulations”:TI,AB,KW OR “student anxiety”:TI,AB,KW OR “classroom anxiety”:TI,AB,KW OR “examination anxiety”:TI,AB,KW)	June 20, 2026
*Biblioteca Virtual em Saúde* or Portal Regional	(TW:(“ansiedade social” OR “ansiedade de desempenho” OR “falar em público” OR “apreensão de comunicação”)) AND (TW:(“estudantes de ciências da saúde” OR “estudantes universitários” OR “estudantes de medicina” OR “estudantes de enfermagem” OR “estudantes de psicologia”)) AND (TW:(“psicometria” OR “validação” OR “instrumentos de avaliação” OR “questionários” OR “escalas” OR “avaliação” OR “medida” OR “ferramenta” OR “instrumento” OR “entrevista clínica” OR “observação” OR “apresentações orais” OR “seminários” OR “prática clínica” OR “simulações” OR “ansiedade do estudante” OR “ansiedade em sala de aula” OR “ansiedade de exame”))	June 20, 2026
LILACS	(ansiedade social[DECS] OR “ansiedade de desempenho” OR “falar em público” OR “apreensão de comunicação”) AND (estudantes de ciências da saúde[DECS] OR “estudantes universitários” OR “estudantes de medicina” OR “estudantes de enfermagem” OR “estudantes de psicologia”) AND (psicometria[DECS] OR “validação” OR “instrumentos de avaliação” OR “questionários” OR “escalas” OR “avaliação” OR “medida” OR “ferramenta” OR “instrumento” OR “entrevista clínica” OR “observação” OR “apresentações orais” OR “seminários” OR “prática clínica” OR “simulações” OR “ansiedade do estudante” OR “ansiedade em sala de aula” OR “ansiedade de exame”)	June 20, 2026
Web of Science	TS=((“social anxiety” OR “performance anxiety” OR “public speaking” OR “communication apprehension”) AND (“health students” OR “medical students” OR “nursing students” OR “psychology students” OR “university students”) AND (“psychometric” *OR* “*validation*” *OR* “*assessment instrument*” OR “questionnaire” *OR* “*scale*” OR “assessment” OR “measurement” OR “evaluation” OR “tool” OR “instrument” OR “rating” OR “clinical interview” OR “observation” OR “oral presentation” *OR* “*seminar*” OR “clinical practice” OR “simulation*” OR “student anxiety” OR “classroom anxiety” OR “examination anxiety”))	June 20, 2026

^a^The strategies were designed to address the potential “indexing gap,” whereby relevant behavioral or educational studies may not be tagged with specific psychometric descriptors. By including terms such as “clinical interview,” “observation,” and “rating,” the search will encompass a wider variety of assessment methodologies. Furthermore, the inclusion of context-specific terms such as “clinical practice” and “simulations” ensures that studies focusing on anxiety during professional training—a critical area for health sciences education—are systematically identified.

The search will be conducted in the following 5 electronic databases: PubMed, Scopus, Embase, LILACS, and Web of Science. Table 2 presents examples of the descriptors and free-text terms, combined with Boolean operators (AND and OR), which will be adapted for each specific database (Table 2).

No temporal restrictions will be applied to the search. The inclusion of studies will be limited to publications in English, Spanish, and Portuguese.

### Inclusion and Exclusion Criteria

The selection of studies will be strictly guided by predefined inclusion and exclusion criteria, developed to directly address the population, concept, and context [21] elements of the research question (Table 3).

**Table 3:** Eligibility criteria based on the population, concept, and context (PCC) framework.

PCC element	Inclusion criteria	Exclusion criteria
Population	Eligible studies will involve undergraduate or postgraduate university students enrolled in health programs (eg, nursing, psychology, medicine, physiotherapy, speech-language pathology, and dentistry). Additionally, studies including mixed populations (health and nonhealth students) will be included only if data specific to health sciences students are clearly disaggregated and separately reported.	Studies deemed ineligible will involve populations outside the health sciences (eg, engineering, law, and arts), unless data for health sciences students are clearly disaggregated and reported separately. Studies in which data for health sciences students cannot be isolated or analyzed separately from those of nonhealth populations will also be excluded.
Concept	Eligible studies will investigate instruments, measures, tools, or assessment approaches used to assess PSA^a^ and CA^b^, specifically in oral communication contexts. Eligible instruments include scales, questionnaires, inventories, rating systems, clinical interviews, observation instruments, self-report measures, and other psychometric tools. Additionally, studies assessing anxiety symptoms (eg, fear, nervousness, and stress) in academic speaking situations will also be included, even if they do not explicitly use psychometric terminology in their title or abstract.	Studies addressing social anxiety in contexts unrelated to oral or public communication (eg, fear of public spaces, informal social interactions, or digital social networks) will be deemed ineligible. Studies focusing broadly on general social anxiety or social phobia without specific reference to oral communication or public speaking will also be excluded. Studies using generic or nonspecific instruments, those that fail to adequately describe the assessment tools used, or those using instruments that have not been validated or for which psychometric properties (eg, reliability, validity, or cultural adaptation) are not reported will likewise be excluded.
Context	Research conducted in academic or health professional training contexts will be eligible for inclusion. These contexts cover scenarios such as oral presentations, seminars, group discussions, clinical simulations, and interactions with real or simulated patients. The review will also include studies examining instrument application in educational settings that promote oral communication skills, such as communication workshops, professional communication training programs, and simulation-based learning environments.	Studies will be deemed ineligible if they do not present empirical data or sufficient methodological details, such as editorials, letters to the editor, and conference abstracts without complete results. Studies will be excluded if they are conducted in nonacademic or nonprofessional health training settings (eg, community-based anxiety disorders clinics or general population samples), unless the sample includes health sciences students and their data are clearly disaggregated and reported separately.
Study type and language	Eligible publications will include original research papers, systematic reviews, instrument validation studies, dissertations, theses, or technical reports providing relevant empirical data. Publications will be limited to those available in English, Spanish, or Portuguese, with no temporal restrictions. Eligible gray literature sources will include institutional dissertations, master’s theses, doctoral theses, and technical reports from universities and research institutions that have undergone institutional review and contain complete methodological descriptions and results.	Ineligible publications will include duplicate studies identified across databases or in multiple versions (only the most complete or recent version will be retained for analysis). Opinion pieces, commentaries, narrative reviews without empirical data, case reports without systematic application of assessment instruments, and conference proceedings lacking full-text availability or sufficient methodological information will be excluded.

^a^PSA: public speaking anxiety.

^b^CA: communication apprehension.

### Data Collection Instrument

A standardized instrument for data collection will be developed and preliminarily tested to ensure consistent and comprehensive extraction of pertinent information. This instrument, presented in tabular format in Multimedia Appendix 1, has been designed to capture all data necessary to answer the research question and meet the established objectives, including details about the study (authors, year, and country), population characteristics, instrument characteristics (type, validation status, and psychometric properties), and application context.

### Study Selection and Data Collection Process

Initially, duplicate records will be systematically removed from the dataset to optimize the screening process. Subsequently, 2 independent reviewers will screen the titles and abstracts of the remaining studies, based on the predefined eligibility criteria, using the Rayyan platform to efficiently manage the results. In situations of disagreement regarding the potential inclusion of a study, a third reviewer will be consulted to arbitrate and determine the study’s relevance to the research question. A complementary manual search will be performed of the reference lists of included studies to identify relevant sources that may not have been captured by the initial database search.

The full texts of papers that advance from the initial screening will be subsequently evaluated by a primary researcher to confirm their full adherence to the inclusion criteria. Reasons for the exclusion of any studies after full-text evaluation will be meticulously documented to ensure methodological transparency. Throughout all phases of the selection process, any discrepancies among team members will be resolved through consensus or, if necessary, with the intervention of an additional reviewer. The complete selection process will be documented and presented using a PRISMA-ScR (Preferred Reporting Items for Systematic Reviews and Meta-Analyses extension for Scoping Reviews) flow diagram to ensure reproducibility and clarity in reporting [16,17].

### Data Analysis

The analysis of evidence will be conducted through a mixed methods approach, integrating quantitative and qualitative techniques for a comprehensive synthesis of the extracted data. Morphological variables, such as authorship, publication year, country of origin, and journal distribution, will be subjected to descriptive statistical analyses, including absolute and relative frequencies and measures of central tendency and dispersion. These analyses will allow the characterization of temporal, geographical, and bibliometric patterns in the literature. Visualization techniques, such as bar charts, line graphs, and heat maps, will be applied to illustrate trends in scientific production and study distribution.

Content-related variables will be examined through a combination of thematic synthesis and, where data homogeneity permits, meta-analysis of psychometric evidence. Studies will be categorized according to instrument type, evaluated psychometric properties, and application context. This categorization will facilitate systematic comparisons and the identification of research gaps. A professional specialized in psychometrics and instrument validation will be actively involved in the analysis, ensuring the robustness and precision of the psychometric data evaluation.

## Results

### Overview

As of May 2026, the preliminary stages of this scoping review have been completed, including the finalization of the search strategy and the selection of electronic databases. The formal literature search across all specified databases is scheduled to commence in June 2026, with the screening of titles and abstracts expected to be finalized by August 2026. The full-text review and data extraction phases are projected to be completed by October 2026, followed by data synthesis and preparation of the final report by December 2026 [22].

The results of this study will be presented in accordance with the PRISMA-ScR. A comprehensive flowchart will be provided to document the flow of information through the different phases of the review, detailing the number of records identified, screened, assessed for eligibility, and ultimately included in the synthesis. Reasons for the exclusion of full-text papers will be explicitly categorized and reported to ensure methodological transparency and reproducibility [17,23].

The final synthesis is expected to provide a multidimensional mapping of the available evidence, categorized by instrument type, target population within the health sciences, and specific psychometric properties evaluated. We anticipate the identification of a heterogeneous range of tools, varying from general social anxiety scales to specific measures of PSA and CA. The findings will be summarized in evidence tables and accompanied by a narrative synthesis that highlights the current state of instrument validation and identifies critical gaps in the literature regarding cultural adaptation and linguistic equivalence in the Brazilian context [24,25].

### Current Timeline Documentation

The protocol specifies the following operational milestones:

- Protocol registration with OSF [18]
- Systematic database searching from June 2026 to December 2026
- Seven-month operational window for comprehensive database searching and gray literature retrieval
- Data extraction and analysis in Q1 (period 1) 2027 (projected)
- Manuscript preparation and dissemination in Q2 (period 2) 2027 (anticipated)

### Study Status

The protocol is currently at the presearch stage, with protocol development and preregistration completed and formal search initiation scheduled for June 2026.

### Expected Study Progression

The study is expected to progress through the following stages:

- Database searching across 7 indexed sources (PubMed, Scopus, Embase, LILACS, Web of Science, the Cochrane Library, and the *Biblioteca Virtual em Saúde* Portal), with concurrent gray literature retrieval
- Dual-reviewer title and abstract screening with an arbitration protocol for disagreements
- Full-text review of potentially eligible studies
- Data extraction using a standardized instrument (Multimedia Appendix 1)
- Mixed methods analysis incorporating descriptive statistics, thematic synthesis, and integration of psychometric expertise

## Discussion

It is hypothesized that, although a significant number of instruments exist to measure social anxiety, there remains a notable scarcity of tools specifically validated for the unique academic and clinical pressures faced by health sciences students. We anticipate that the findings will reveal a conceptual overlap between general social phobia and the more specific constructs of PSA and CA, often leading to diagnostic imprecision. Furthermore, it is expected that many studies rely on instruments that lack robust cross-cultural validation, potentially limiting their applicability across diverse educational systems [14,26].

The characterization of these instruments is paramount for the advancement of health education. By distinguishing between task-specific anxiety (PSA) and broader communication-related fears (CA), educational institutions can move beyond generic support and implement targeted pedagogical scaffolding. The precision in assessment enabled by validated tools is essential for identifying students who may require specific interventions to develop clinical communication competencies, which are directly linked to patient safety and the quality of the therapeutic relationship [27,28].

Beyond the academic sphere, the implications for student mental health are profound. Early and accurate identification of high levels of CA can prevent the development of maladaptive coping mechanisms and reduce the risk of academic attrition. From a professional training perspective, fostering communicative resilience through evidence-based assessment ensures that future health professionals are better equipped to handle high-stakes interactions, such as breaking bad news or coordinating within multidisciplinary teams. Therefore, this scoping review will serve as a foundational evidence base for future research focused on the longitudinal impact of PSA on professional identity and clinical performance [29,30].

## Appendix

Multimedia Appendix 1 Data extraction instrument.

## Abbreviations

CA: communication apprehension
MeSH: Medical Subject Headings
OSF: Open Science Framework
PRISMA-ScR: Preferred Reporting Items for Systematic Reviews and Meta-Analyses extension for Scoping Reviews
PSA: public speaking anxiety
