# Supplementary material for: Assessment Instruments for Social Anxiety in Oral and Public Communication Among Health Sciences Students: Protocol for a Scoping Review
Source: JMIR Res Protoc. 2026 Jun 29;15:e93700. doi: 10.2196/93700 (PMC13365892; doi:10.2196/93700)
Supplement: Multimedia Appendix 1 [file resprot_v15i1e93700_app1.docx]

**Multimedia Appendix 1: Data Extraction Instrument**

This appendix provides the template for data extraction from the included studies. Data will be systematically collected using this instrument in Microsoft Excel.

**Data Extraction Form**

| **Category** | **Variable** | **Description / Format** | **Example / Notes** |
| --- | --- | --- | --- |
| **Morphological Variables** | Study ID | Unique identifier for each record. | e.g., Study_001 |
|  | Article Title | Full title of the publication. |  |
|  | Authors | Full list of authors, in order. | e.g., Smith J, Doe A, Brown B |
|  | Year of Publication | Year the article was published. | e.g., 2023 |
|  | Country of Origin | Country where the study was conducted or the primary author's affiliation. | e.g., Brazil, USA |
|  | Journal Name | Full name of the journal (if applicable). | e.g., Journal of Health Sciences Education |
|  | Volume | Journal volume number. |  |
|  | Issue | Journal issue number. |  |
|  | Page Numbers | Starting and ending page numbers. | e.g., 123-145 |
|  | Digital Object Identifier (DOI) | Unique alphanumeric string for digital content. | e.g., 10.xxxx/xxxx.xxxx |
| **Content-Related Variables** | Type of Publication | Category of the publication. | e.g., Original Research, Validation Study, Systematic Review, Dissertation, Thesis, Technical Report |
|  | Instrument Name | Full name of the assessment instrument used. | e.g., Liebowitz Social Anxiety Scale, Fear of Public Speaking Questionnaire |
|  | Year of Instrument Validation | Year the instrument was initially validated or culturally adapted (if specified). | e.g., 2010 |
|  | Instrument Type | Specific type of assessment tool. | e.g., Scale, Questionnaire, Inventory |
|  | Psychometric Properties Evaluated | Key psychometric properties assessed in the study. | e.g., Reliability (internal consistency, test-retest), Validity (construct, convergent, discriminant), Factor structure |
|  | Focus of Social Anxiety Assessment | Specific aspect of social anxiety measured. | e.g., General social anxiety, Performance anxiety, Fear of public speaking, Communication apprehension |
|  | Study Design | Methodological design of the study. | e.g., Cross-sectional, Longitudinal, Experimental, Quasi-experimental |
|  | Sample Size | Number of participants in the study. |  |
|  | Academic Context of Application | Specific academic scenario where the instrument was applied. | e.g., Classroom presentations, Clinical simulations, Patient interaction, Group discussions |
|  | Mode of Administration | How the instrument was administered. | e.g., Paper-based, Digital (online), Observational, Interview |
| **Cultural & Outcomes Variables** | Cultural or Linguistic Adaptation Details | Information on adaptation for different cultures/languages. | e.g., Adapted for Brazilian Portuguese, cross-cultural validation performed |
|  | Reported Outcomes / Instrument Performance | Key findings regarding the instrument's performance or applicability in the study. | e.g., High reliability, good validity, limitations noted, specific cutoff scores |
